# Towards extracellular matrix normalization for improved treatment of solid tumors

**DOI:** 10.7150/thno.39995

**Published:** 2020-01-12

**Authors:** Hoda Soleymani Abyaneh, Maximilian Regenold, Trevor D. McKee, Christine Allen, Marc A. Gauthier

**Affiliations:** 1 Institut National de la Recherche Scientifique (INRS), EMT Research Center, 1650 boul. Lionel-Boulet, Varennes, J3X 1S2, Canada.; 2 Leslie Dan Faculty of Pharmacy, University of Toronto, 144 College Street, Toronto, Ontario M5S 3M2, Canada.; 3 STTARR Innovation Centre, University Health Network, 101 College Street Room 7-504, Toronto, Ontario M5G 1L7, Canada

**Keywords:** tumor extracellular matrix, collagen, hyaluronic acid, fibrosis, nano-formulations

## Abstract

It is currently challenging to eradicate cancer. In the case of solid tumors, the dense and aberrant extracellular matrix (ECM) is a major contributor to the heterogeneous distribution of small molecule drugs and nano-formulations, which makes certain areas of the tumor difficult to treat. As such, much research is devoted to characterizing this matrix and devising strategies to modify its properties as a means to facilitate the improved penetration of drugs and their nano-formulations. This contribution presents the current state of knowledge on the composition of normal ECM and changes to ECM that occur during the pathological progression of cancer. It also includes discussion of strategies designed to modify the composition/properties of the ECM as a means to enhance the penetration and transport of drugs and nano-formulations within solid tumors. Moreover, a discussion of approaches to image the ECM, as well as ways to monitor changes in the ECM as a function of time are presented, as these are important for the implementation of ECM-modifying strategies within therapeutic interventions. Overall, considering the complexity of the ECM, its variability within different tissues, and the multiple pathways by which homeostasis is maintained (both in normal and malignant tissues), the available literature - while promising - suggests that improved monitoring of ECM remodeling *in vivo* is needed to harness the described strategies to their full potential, and match them with an appropriate chemotherapy regimen.

## Introduction

Most chemotherapy regimens involve the systemic administration of cytotoxic drugs and are often associated with dose-limiting toxicities due to off-target effects. To address this concern, novel nano-formulations that rely on nano-sized particles or entities formed from organic (e.g., polymer or lipid) or inorganic materials (e.g., gold) have been developed for drug delivery. These formulations alter the pharmacokinetics of the drug and exploit the unique tumor biology in a manner that promotes accumulation at the tumor site, rather than in healthy tissue 1. This approach has led to reductions in side effects, with less prominent improvements in treatment efficacy and clinical outcomes 2. This is in part due to obstacles that prevent the homogenous distribution of drugs and nano-formulations throughout the bulk of solid tumors 3. Although some physical characteristics of tumors promote the accumulation and retention of nano-formulations at the site of the tumor, the same and other characteristics can restrict convective as well as diffusive transport of these systems within the tumor itself. More specifically, drug accumulation and retention in tumors can be promoted by the leaky vasculature and impaired lymphatic drainage present within some solid tumors, leading to a phenomenon known as the enhanced permeability and retention (EPR) effect 4. During tumorigenesis, the increased secretion of vascular growth factors leads to formation of abnormal vasculature with intercellular gaps and endothelial fenestrae that contribute to an overall leakiness of these vessels 5. For instance, the tumor vessel basement membrane has a heterogeneous thickness, is often loosely associated with endothelial cells and has reduced pericyte coverage compared to healthy vessels 6. Pericytes are perivascular cells that lie within the basement membrane, structurally stabilize the endothelium, and interact with endothelial cells 7. They are mainly responsible for reducing endothelial cell proliferation, which explains why tumor vessels often lack pericyte coverage 8, 9. Moreover, in the case of low pericyte coverage the shedding of cancer cells from the tumor is increased since pericytes are known to prevent breach of the vessel wall 10. These vasculature abnormalities result in the formation of pores that are 100 times larger than those in healthy vessels 11 and thus permit the extravasation of nanoparticles below 400 - 600 nm in size from blood into the tumor 12. While this can be seen as advantageous for promoting the accumulation of nano-formulations, these phenomena also result in decreased total length and penetration of blood vessels within the tissue volume, leading to reduced blood flow 13. This causes some tumor areas to be poorly perfused and inaccessible to nano-formulations, whose primary mode for tumor delivery relies on travelling within the systemic vasculature 14. Once delivered to the tumor site, the predominant mechanism underlying nano-formulation transport into the tumor mass is convection, driven by hydrostatic pressure gradients between the tumor microvasculature and the tumor interstitium 15.

While in normal tissue, a balance exists between blood flow and lymphatic drainage, many solid tumors lack functional lymphatic drainage due to a number of mechanisms, resulting in reduced clearance of interstitial fluid containing biomolecules, immune cells, or nano-formulations 16, 17. The combination of enhanced vascular permeability and an absence of functional lymphatics thus leads to an elevated interstitial fluid pressure exhibiting a complex gradient throughout the tumor mass compared to healthy tissue 18. Along the tumor periphery, a functioning lymphatic system reduces the interstitial fluid pressure, resulting in a pressure gradient that promotes convective transport towards the tumor mass. This leads to the accumulation of nano-formulations mostly along the tumor periphery 18-20. However, within the core of the tumor mass, the interstitial fluid pressure is significantly elevated, which in particular impedes the homogeneous distribution of nano-formulations throughout the tumor volume 14, 19-23. Thus, within these areas, diffusion is the main driving force for transport of nano-formulations 22. For similar reasons, it is particularly difficult for nano-formulations to reach hypoxic regions, which are mostly found within the poorly perfused core of the tumor and are commonly associated with abnormal vasculature, resulting in a decreased supply of oxygen, nutrients, and drugs to these regions 24, 25. Many strategies have been tested to overcome this challenge, such as by developing hypoxia- or pH-specific nano-formulations 26, 27. Another strategy to overcome this challenge is vessel normalization. It has been shown that direct or indirect blockade of vascular endothelial growth factor (VEGF) signaling pathways with therapeutic agents have the potential to repair vessel disorganization (i.e., vessel normalization) 6, 7. Of note, this approach is different from traditional anti-angiogenic strategies that aim to reduce the total number of immature and mature vessels by destroying existing vessels and/or inhibiting the formation of new vessels as a means to starve the tumor 28. The aim of vessel normalization is to decrease tumor interstitial fluid pressure, increase perfusion and oxygenation, and sustain vessel normalization by reducing the number of some immature vessels while increasing the maturity of the average remaining vessels 6, 7. This approach is expected to increase the exposure of cancer cells to anticancer therapies as well as to decrease the number of hypoxic areas responsible for tumor progression and metastasis 7.

In addition to the features discussed above, the dense and aberrant extracellular matrix (ECM) of solid tumors is another major contributor to the heterogeneous distribution of nano-formulations within tumors 29. The combined forces of dividing tumor cells, and the production and deposition of ECM components (such as collagen and hyaluronic acid), leads to the presence of high solid stress in tumors 18, 30. Solid stress is distinct from the interstitial fluid pressure. The interstitial fluid pressure is derived from the leaky vasculature causing equilibration of intra- and extra-blood vessel pressures within tumor. Solid stress arises from mechanical forces of the solid phase of the tumor. Components of the tumor tissue contributing to solid stress include dividing tumor cells, and the combination of forces arising from collagen, which has high tensile properties, and hyaluronic acid and glycosaminoglycans, which exert a gelation pressure due to retention of fluid. The combination of these forces can collapse blood vessels in tumors, further impeding drug delivery 31.

ECM normalization, similar to vessel normalization, is a new approach that focuses on remodeling the microenvironment to resemble that of healthy tissue rather than complete destruction of the ECM components 7, 32. It is suggested by Von Hoff et al., that this approach will be most successful with therapeutic agents that have overall effects on transcription and cellular reprogramming 32. This contribution presents the current state of knowledge regarding the composition of normal ECM, changes to ECM that occur during the pathological progression of cancer, and strategies designed to modify its composition/properties to enhance the tumor penetration and interstitial diffusion of drugs and nano-formulations within solid tumors. Particular emphasis will be placed on the non-cellular components of the ECM, and the reader is referred to other recent reviews for a discussion on targeting cellular components such as fibroblasts or immune cells 33-36.

## ECM components and their roles

**Figure 1:**
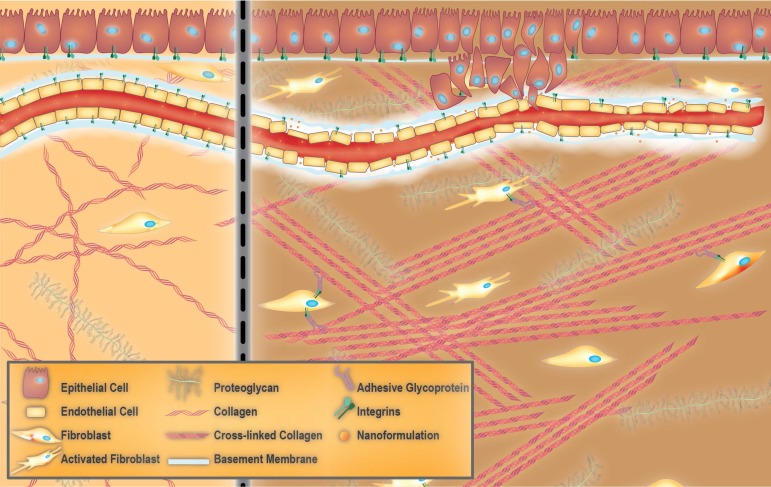
** Towards extracellular matrix (ECM) normalization for improved treatment of solid tumors.** Healthy ECM versus aberrant tumor ECM (left and right panels, respectively). Healthy ECM is characterized by the presence of an intact basement membrane, non-activated fibroblasts and random arrangement of collagen fibers (left panel). Aberrant tumor ECM features the tumor vessel basement membrane with a heterogeneous thickness that allows the dissemination of tumor cells as well as accumulation of nano-formulations. The presence of collagen fibers which are aligned in an ordered fashion and activated fibroblasts are other characteristics of tumor ECM.

The ECM is a complex biomaterial that exists between clusters of cells in all tissues 37-39, and is often interchangeably referred to as the interstitial matrix, or the acellular portion of the stroma. Fundamentally, the ECM consists of polysaccharides, proteins, and water (**Figure 1**). It provides mechanical support for cells, as well as biochemical and physical cues, which are necessary for tissue development, differentiation, and homeostasis 38, 39. All ECM constituents are produced by the various cell types residing within the scaffold, including fibroblasts, epithelial cells, endothelial cells, and immune cells 40. However, the composition of the ECM and its structure can vary significantly between tissues (e.g., kidneys vs. liver), within one tissue (e.g., renal cortex vs. renal medulla), and between different physiological states (e.g., normal vs. pathological) 38. These differences in composition and structure of the ECM also exist among species (humans versus rodents). Rodents, notably mice, are commonly used as models of multiple human diseases. This is mostly due to the high degree of similarity in the sequences of genes between humans and mice 41, 42. However, minor differences in their genetic makeup may cause profound differences in cellular development. For instance, genes responsible for normal collagen I fibrillogenesis such as *collagen, type III, alpha-1* (*COL3A1*) are known to be instrumental in development and function of the ECM of the lung. However, the *COL3A1* gene network and regulation are different between humans and mice, which complicates the use of mouse models to study certain types of human lung diseases 43. Another example of such a discrepancy is a higher expression of the ECM components in the human brain compared to that of the mouse. This evolutionary expansion of the human brain leads to higher cognitive function 44. Fortunately, newly developing proteomic and computational approaches have significantly helped in understanding and characterizing the differences in ECM composition of healthy and diseased tissue in humans as well as in model organisms (i.e., matrisome project) 45.

Organs are divided into stromal and parenchymal constituents based on histology. The parenchymal component is the part of the organ that completes its function, such as myocardial cells in the heart or hepatocytes in the liver. The parenchyma is surrounded by the stromal compartments of the organ such as blood vessels, nerves, and connective tissue 46. For any given tissue, a basement membrane separates the parenchyma from the stroma 37, 38. The ECM within the basement membrane is biochemically and structurally distinct from the mesenchymal/interstitial stromal ECM (hereafter referred to as stromal ECM for the sake of simplicity) (**Figure 1**) 47. Mesenchyme, also known as mesenchymal tissue, refers to a group of cells which are derived from the mesoderm 48. Mesenchymal cells (such as fibroblasts) are responsible for the development of haematopoietic and connective tissues such as the bone marrow, bones, cartilage, muscles, tendons, and ligaments 48, 49.

### The basement membrane

When the basement membrane was first visualized by transmission electron microscopy, it was considered to be similar to stromal ECM 37. However, it was later realized that the basement membrane was more compact and less porous than stromal ECM, and was always associated with cells 37, 39. Thus, the basement membrane can be considered a specialized ECM-like material that is associated with epithelial and endothelial cells lining blood vessels 37, 49. All cells within a tissue produce basement membrane constituents. However, the molecular composition of the basement membrane is unique to each tissue. This biochemical variability is considered to provide the cellular microenvironment necessary for conferring specific functionality to tissues.

### Cellular components of stroma

**Figure 2:**
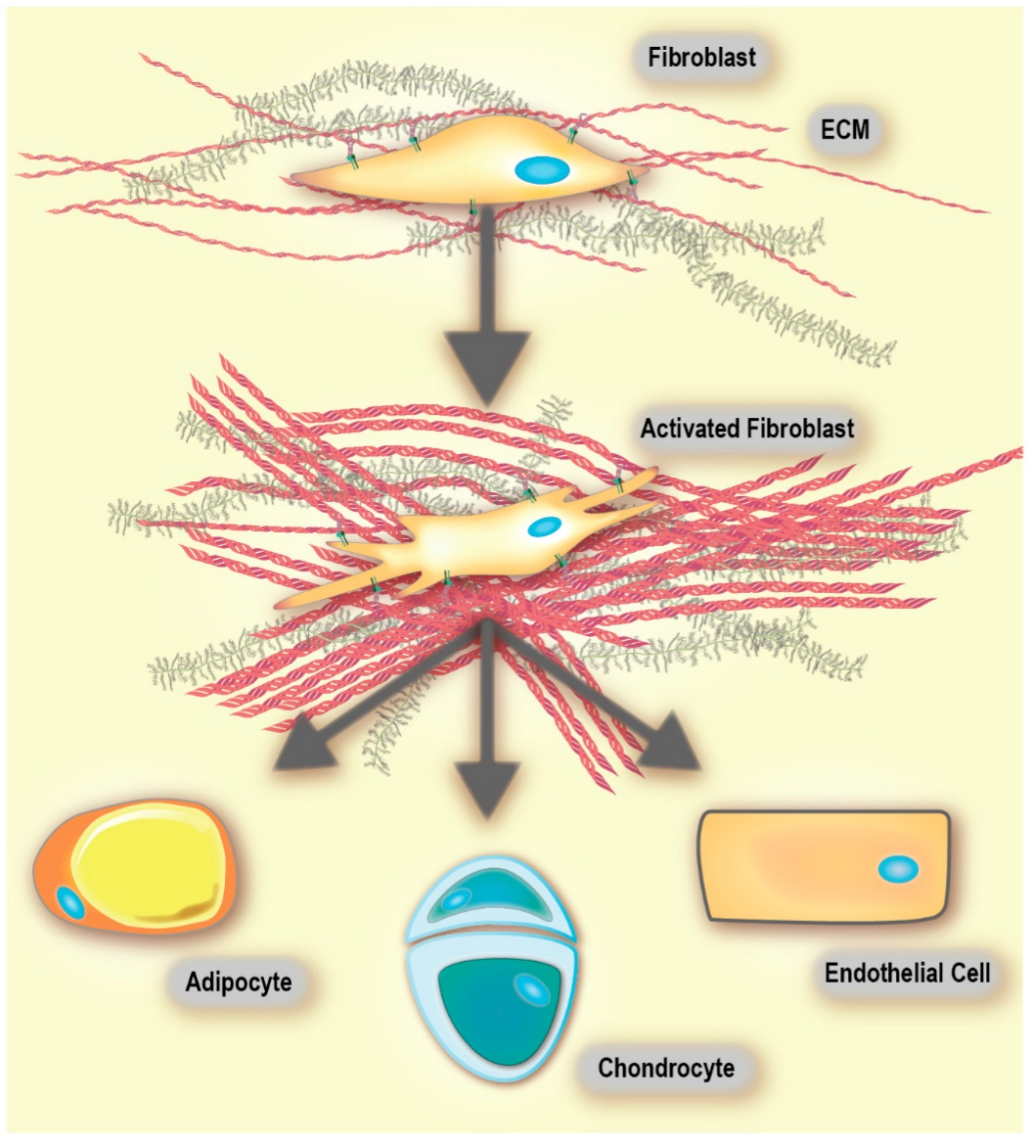
** Fibroblasts are highly plastic and exhibit multi-potency.** Activated fibroblasts readily differentiate into adipocytes, chondrocytes and endothelial cells, among others. Adapted with permission from 49, Copyright 2016 Springer Nature. Of note, there are conflicting reports on the differentiation of activated fibroblasts into adipocytes 197.

Virchow, and later Duvall's first reports of cells within connective tissue were published in the mid-19th century. Later, these cells were named fibroblasts and found to produce collagen 49. Fibroblasts are non-immune, non-epithelial cells, originating from the mesenchyme and exhibit a spindle-shaped morphology 49, 50. In healthy tissue, they are mostly found as non-activated isolated cells within the stromal ECM. However, non-activated fibroblasts have the ability to become activated when needed 49. When comparing fibroblasts derived from either healthy tissue or a healing wound, the latter have been found to produce larger amounts of ECM and proliferate faster 49, 51. These fibroblasts are called activated 52, and are responsible for secretion of chemokines and cytokines, recruitment of immune cells, production of ECM components and enforcing mechanical control over the tissue structure (*vide infra*) 49, 53, 54. Activated fibroblasts are commonly called myofibroblasts due to the expression of α‑smooth muscle actin (αSMA), which is a cytoskeletal protein found in smooth muscle cells 49, 55. An activated fibroblast also has the ability to differentiate into other cell types of the mesenchymal lineage, including chondrocytes (primary cell type of cartilage 56), adipocytes (fat cells), and endothelial cells (**Figure 2**). This is because non-activated fibroblasts possess characteristics that are similar to mesenchymal stem cell (MSC) precursors. MSCs are multipotent stromal cells that have the ability to differentiate into different types of cells that form the connective tissue of many organs. Thus, as suggested by Kalluri, a non-activated fibroblast can be thought of as an adult tissue-resident mesenchymal stem cell 49.

### ECM proteins, glycoproteins, and proteoglycans

In contrast to the basement membrane, the bulk of the stromal ECM is rich in fibrous proteins, glycoproteins, and proteoglycans (**Figure 1**). Due to the highly charged and hydrated nature of the stromal ECM, it confers resistance towards compressive stressors to the tissue 39, 57. The most prevalent fibrous protein within the stromal ECM is collagen. The collagen family contains at least 28 distinct types, with types I and III being the most commonly found 58, 59. Most collagens self-assemble into triple-helical structures. However, the type of collagen determines the overall supramolecular organization into structures such as fibrils and networks 38. Fibers consist of a heterogeneous mixture of various collagen types, although tissues commonly contain only one type. Cross-linking can occur either intra-molecularly (i.e., within the triple helix) or inter-molecularly (i.e., between neighbouring triple helices), and is often assisted by the presence of non-helical telopeptides in the NH_2_ and COOH regions of the collagen molecule 60, 61. Fibril bundles within the stromal ECM are composed of fibrous collagens, while network collagens are integrated into the basement membrane 38. Fibrillar collagens are responsible for providing tensile strength, whereas network collagens (e.g., collagen IV) are essential for connecting the ECM to the vasculature 40, 62. Elastin is another important fibrous ECM protein. It provides tissues that are frequently stretched with the elasticity required to maintain such functions. Secreted tropoelastins, precursors of elastin, assemble into fibers and become highly cross-linked to one another by the lysyl oxidase (LOX) enzyme family. LOXs are mainly responsible for cross-linking ECM components such as collagen and elastin, which results in stiffening of the ECM 38, 63. The cooperation between elastin and collagen plays a crucial role in limiting the extent of elastin stretching 38, 64.

The other major class of macromolecules in stromal ECM are proteoglycans 65, 66. Most proteoglycans are composed of glycosaminoglycan (GAG) chains that are connected to a protein core by a covalent bond with the exception of hyaluronic acid that is present in its free form 66, 67. GAGs are long unbranched polysaccharide chains that contain repeating disaccharide units composed (except for keratan) of a galactose or an uronic sugar (D-glucuronic or L-iduronic acid) along with an amino sugar (N-acetylgalactosamine or N-acetylglucosamine). These polymers are often sulfated, which introduces a high negative charge and structural heterogeneity (e.g., heparan sulfate, keratan sulfate, and chondroitin sulfate). An example of a non-sulfated GAG is hyaluronic acid 66. Moreover, proteoglycans consist of different types of GAG chains with varying length and composition, adding to their heterogeneity. They are very hydrophilic and can assume extended conformations that allow them to form hydrogels. The interaction of the hydrated GAG network with fibrous ECM molecules is responsible for the resistance of tissues to compressive stressors 68. Moreover, like collagen, proteoglycans demonstrate the ability to bind and store bioactive molecules such as cytokines and growth factors, essentially making them a reservoir of these molecules within the stromal ECM 40, 69.

### Cellular adhesion & ECM modification in the stroma

ECM molecules interact with cells by binding to cell surface receptors such as integrins, cell-surface proteoglycans, glypicans, syndecans, discoidin domain receptors, as well as hyaluronic acid receptors CD44 (cluster of differentiation 44) and RHAMM (receptor for hyaluronic acid-mediated motility). The adherence of cells to the cell-surface receptor is commonly mediated through adhesive glycoproteins such as entactins (or nidogens), fibronectin, fibrinogen, laminins, tenascins, thrombospondins, vitronectin, nephronectin, and others 70. This interaction activates intracellular signaling pathways that subsequently control a myriad of cellular functions 58, 71. It also acts as a physical link between the interior and the exterior of a cell, which enables bidirectional sensing of signals that control cell fate and function(s) for maintaining tissue homeostasis. Tissue homeostasis is heavily dependent on active ECM remodeling, which constantly happens via the dynamic equilibrium of ECM production and degradation under both physiological and pathological conditions. LOXs are mostly responsible for cross-linking and stiffening the ECM 63, while various other families of digestive enzymes are involved in ECM breakdown. ECM degrading enzymes include: a) proteases such as matrix metalloproteinase (MMP), a disintegrin and metalloproteinase (ADAM), ADAM with thrombospondin motifs (ADAMTS), cathepsin, plasminogen activator, and b) GAG-degrading enzymes like hyaluronidase and heparanase (that cleave hyaluronic acid and heparan sulfate chains, respectively) 72, 73. There also exist tissue inhibitors of metalloproteases (TIMPs), a family of endogenous proteins that modulate MMP and ADAM activity in healthy and diseased tissues 74, 75. Upon ECM degradation, matrix-stored growth factors and cytokines are released. These released molecules can then act on the cell surface receptors of ECM-resident cells to modulate their functions for maintenance of tissue homeostasis 40, 72.

## Dysregulation of ECM homeostasis in pathologic conditions

**Figure 3:**
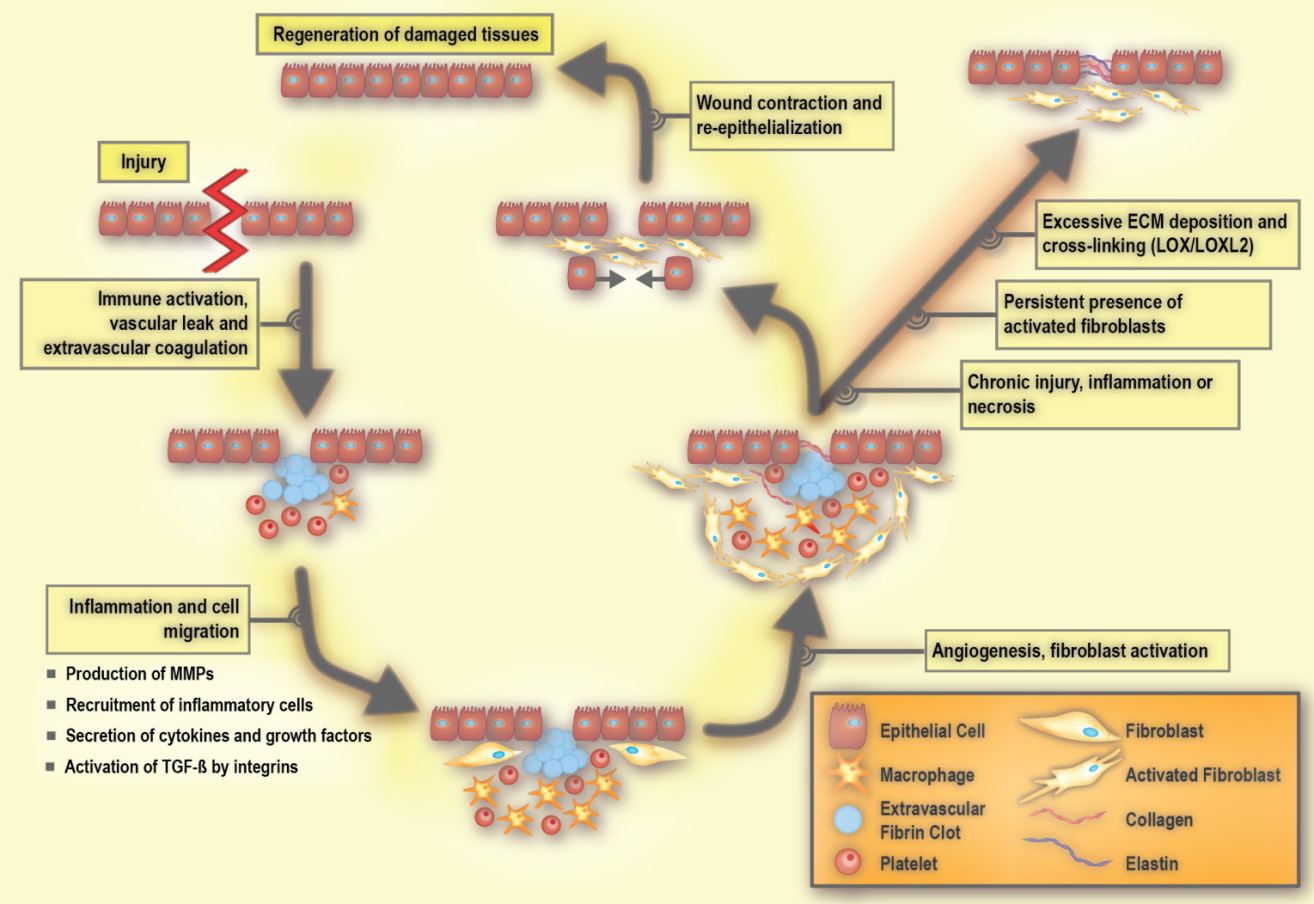
** Molecular processes involved in wound repair and fibrosis**. LOX: lysyl oxidase, LOXL2: lysyl oxidase like 2, MMP: matrix metalloproteinase, PDGF: platelet-derived growth factor, and TGF-β: transforming growth factor beta. Adapted with permission from 198, Copyright 2007 American Society for Clinical Investigation.

Homeostasis in a healthy tissue depends on crosstalk between parenchymal cells and cells in the surrounding stroma, which would primarily be composed of non-activated fibroblasts, adipocytes, and non-stimulated immune cells existing in the steady-state 38, 76. Under such conditions, non-activated tissue fibroblasts produce and organize type I and III collagens, elastin, and various proteoglycans including hyaluronic acid. As such, they maintain the functional and structural integrity of the stromal ECM. In the case of tissue injury, the classic wound healing response involves inflammation and the recruitment of immune cells and fibroblasts to promote angiogenesis as well as the production of ECM (**Figure 3**) 38, 49.

**Figure 4:**
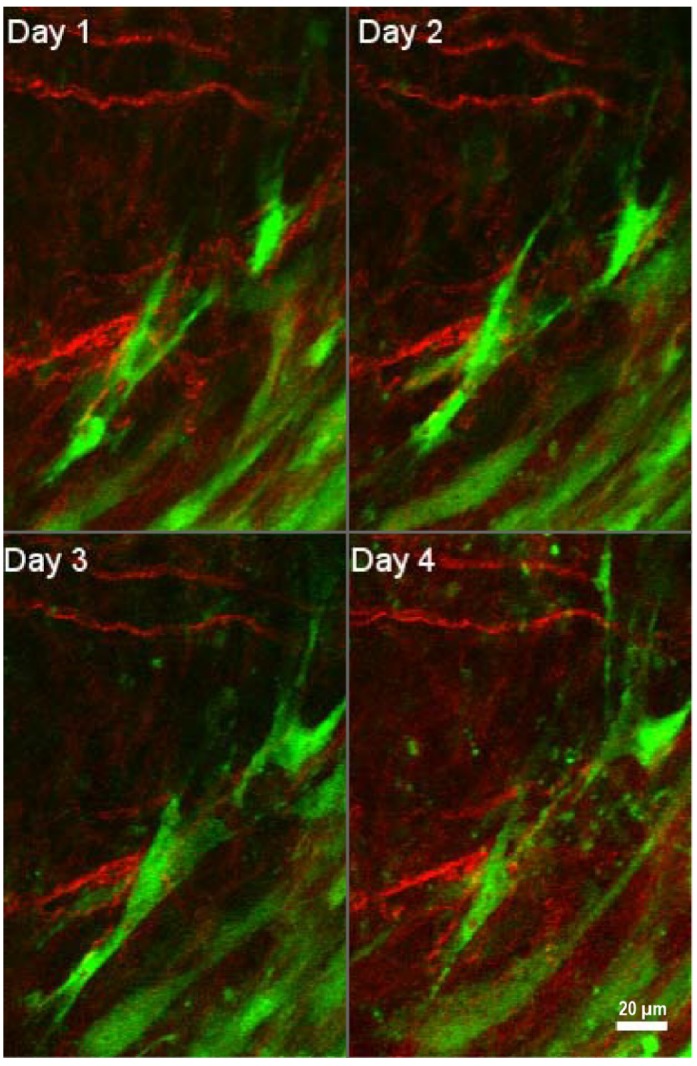
** Interactions of tumor-associated fibroblasts and collagen.** Daily multiphoton laser scanning microscopy images were acquired in a tumor growing in a dorsal skinfold window chamber. Two channels were acquired: Second harmonic generation (SHG) signal arising from collagen (shown in red), and green fluorescent protein (GFP) present in cancer associated fibroblasts (shown in green). Image montage presents a maximum intensity projection of a few images each, acquired 24 hours apart for four consecutive days. Fibroblasts are seen to migrate within the tumor to varying degrees, and occasionally interact with collagen fibers. Figure is generated from data provided by Dr. Trevor D. McKee which was originally captured and analyzed in 181. With permission from 181, Copyright 2009 Springer Nature.

The early process of the wound response includes activation of the coagulation cascade that results in the formation of a fibrin clot in order to seal the vascular damage and prevent infection 38, 40. A subsequent event is the inflammatory response, which includes the production and secretion of growth factors and cytokines, as well as the recruitment of inflammatory cells 40. During inflammation, immune cells including granulocytes and neutrophils are first recruited to the site of the wound, and are then followed by mast cells, lymphocytes, and macrophages. These immune cells release cytokines and chemokines that mobilize fibroblasts to the periphery of the wound 77, 78. In response to these stimuli, the non-activated fibroblasts become activated in order to repair and regenerate the wounded area. Activated fibroblasts are able to produce large amounts of ECM components, including hyaluronic acid, and collagen type I and III (**Figure 4**). Such extensive ECM production and remodeling induces the differentiation of other tissue-resident cells, such as epithelial cells, into activated fibroblasts via an epithelial to mesenchymal transition (EMT) 79, 80. During the EMT, a polarized epithelial cell acquires a mesenchymal phenotype. This phenotype enables the cell to move and gain access to distal sites. An endothelial cell can also undergo a similar process and acquire a mesenchymal cell phenotype via the endothelial to mesenchymal transition (EndMT) 81, 82. During the wound healing process, integrin receptors allow the ECM molecules to interact with cells. Integrins not only contribute to adhesion, but also play an active role in intracellular signaling 83. They facilitate communication between ECM, parenchymal cells, and non-parenchymal cells including inflammatory cells and activated fibroblasts 78, 84. Integrin deficiency inhibits the activation of fibroblasts, resulting in delayed wound closure 78, 85.

Once a wound is repaired, strict feedback mechanisms are in place to ensure that tissue homeostasis is restored and fibrosis is resolved 79, 80. During the resolution of fibrosis, ECM-remodeling enzymes reduce the volume of fibrotic matrix, in particular through the competing activities of LOXs and MMPs. Of note, some members of the MMP family are pro-fibrotic 86. Activated fibroblasts are a main source of MMPs 87 and LOXs 88, underlining their key role in maintaining ECM homeostasis. Once the wound healing is completed, there is a significant reduction in the number of activated fibroblasts due to either apoptosis or reprogramming, which restores them to their non-activated phenotype 49.

Tissue or organ fibrosis, also known as chronic tissue wound healing, is an unresolved form of a wound maintained in part due to persistent inflammation 81. In pathology, fibrosis is referred to as scarring that can be commonly visualized by different histological stains (often leading to ECM band-like patterns that resemble a scar) 49. The imbalance between ECM production and degradation due to the persistent presence of activated fibroblasts results in fibrosis 72. If the insult is perpetual, activated fibroblasts may adopt further secretory phenotypes, an enhanced ability to remodel ECM, and increase their immunomodulatory signalling functions. An unabated wound can help promote the propensity to evolve into a cancerous tumor phenotype 89, 90. Therefore, the tumor stroma shares some of the features that are characteristic of a chronic wound 83, 91.

## 'Cancer is an unresolved wound'

As first suggested by Dvorak, cancer behaves similarly to an unresolved wound 92. The constituents of tumor stroma include the basement membrane, capillaries, activated fibroblasts, immune cells, and ECM surrounding the cancer cells 49, 76. Activated fibroblasts are a main cellular component of the tumor stroma and play a prominent role in promoting tissue desmoplasia (the abundance of collagenous stroma surrounding the tumor 93) to facilitate cancer progression 89, 94. Note that the desmoplastic reaction, tumor stroma, and tumor microenvironment are used interchangeably 49, 83. Fibroblasts associated with cancer are referred to as tumour-associated fibroblasts (TAFs), cancer-associated fibroblasts (CAFs), cancer-associated mesenchymal stem cells, activated myofibroblasts, and activated fibroblasts. On the other hand, activated fibroblasts associated with chronic tissue fibrosis are termed fibrosis-associated fibroblasts (FAFs). At the cellular level, FAFs and CAFs are very similar and potential functional differences at the molecular level, remain to be determined 49.

Fibroblasts are able to exert tension on the matrix and thus can significantly re-organize the structure of collagen fibers. In a healthy tissue, the arrangement of ECM components is random, whereas in a desmoplastic stroma, ECM fibers are aligned in an ordered fashion 47, 95. Activated fibroblasts deposit abundant quantities of ECM proteins and secrete MMPs, growth factors, and cytokines to remodel the ECM 89, 94. As discussed previously, the release of growth factors such as VEGF promotes new vessel growth and enhances vascular permeability, leading to increased interstitial pressure. Tumor growth and impaired lymphatic drainage further contribute to the high interstitial fluid pressure within the tumor mass. This causes an outward flow of fluid from the tumor core to the periphery, facilitating dissemination of cancer cells from the primary tumor 17. Overall, the ECM re-organization, along with solid stress and high interstitial fluid pressure promote tumor progression and metastasis. Notably, the migration of cancer cells towards the vasculature may happen along tension-oriented collagen fibers 96.

**Figure 5:**
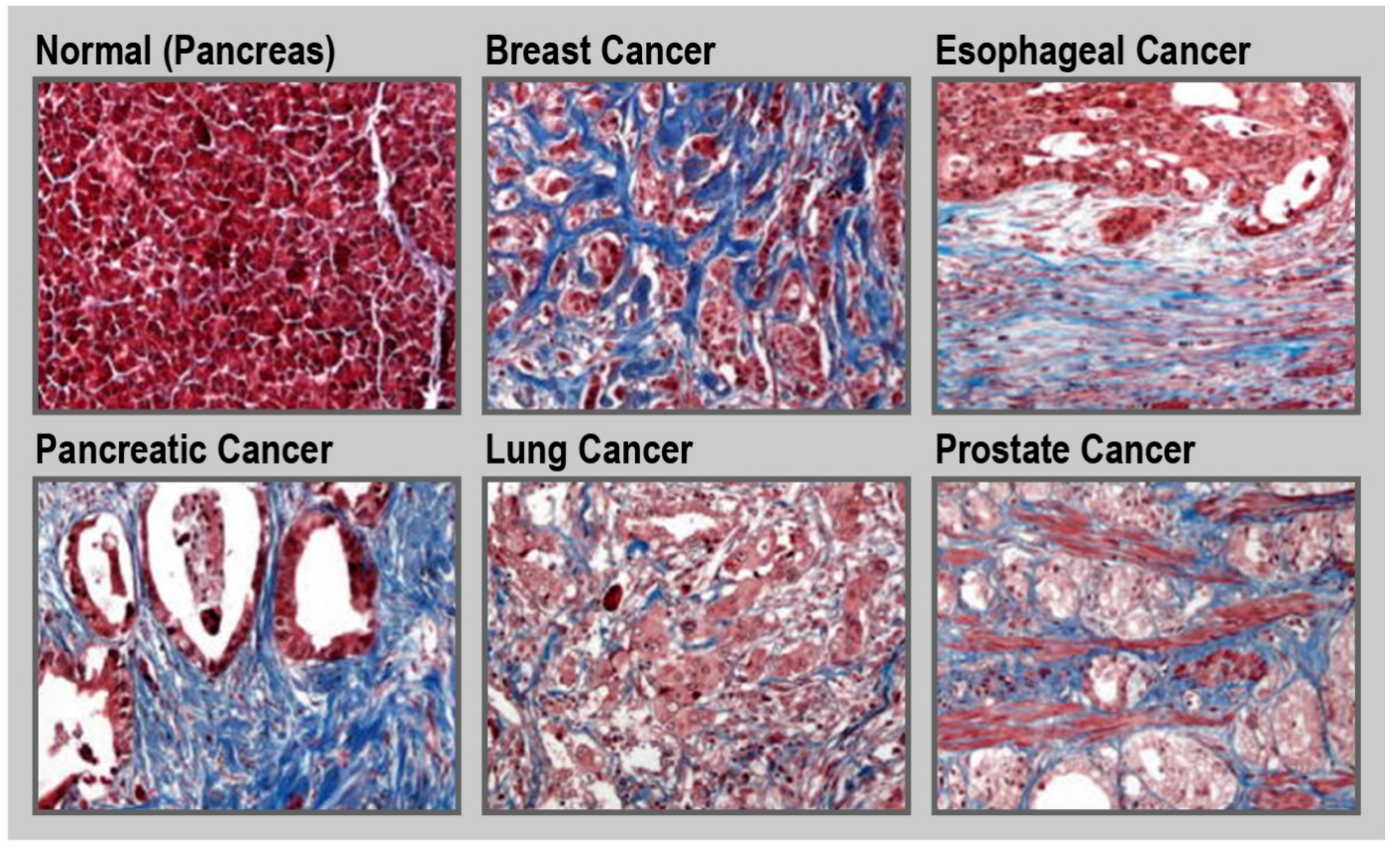
** Fibrosis in cancer.** Representative trichrome (blue) staining for collagen in normal pancreas and different cancerous tissues. Magnification ×20. Reprinted with permission from 34, Copyright 2017 Springer Nature.

Tumor tissue is generally stiffer compared to its healthy counterpart 97. This is due to the increased production of ECM by activated fibroblasts along with an increased contractility of the altered epithelium. Such a fibrotic response is an important feature of certain cancers, including pancreatic cancer, esophageal cancer, prostate cancer, colon cancer, lung cancer, ovarian cancer, and some subsets of breast cancer (**Figure 5**) 30, 34, 98-100. For instance, in the case of pancreatic cancer, the fibrotic stroma can make up to over 80% of the total tumor volume 101, 102. Unfortunately, in general, treatment and diagnosis of fibrosis are limited. Currently, only a few drugs are approved to treat fibrotic diseases. Pirfenidone is a small-molecule that inhibits the transforming growth factor beta (TGF-β) signaling pathways, and is approved to treat idiopathic pulmonary fibrosis 103, 104. Nintedanib, is a small-molecule tyrosine kinase inhibitor that blocks the action of fibroblast growth factor (FGF), platelet-derived growth factor (PDGF), and VEGF that is also used for treatment of idiopathic pulmonary fibrosis 105. Moreover, there are limited clinical methods to monitor disease progression with the detection and diagnosis of fibrosis being mainly dependent on tissue sampling. Therefore, there is a need to design new therapeutic and diagnostic agents for fibrotic conditions 106, 107. For example, ECM homeostatic disruption in radiation-induced skin fibrosis can be assessed in part by monitoring and imaging metabolic changes in dermal fibroblasts 108. Due to the central role of ECM components in fibrosis, these molecules can be attractive pharmacological targets for both therapeutic and diagnostic purposes.

## Collagen as a therapeutic target to remodel ECM

Collagen is the major structural component of the ECM and its overproduction/deposition is a significant contributor to fibrosis. Thus, there has been great interest in developing therapeutic strategies that target the collagenous component of the ECM. These approaches are classified as: (1) inhibition of collagen synthesis; (2) degradation of stromal collagen; (3) inhibition of collagen cross-linking; and (4) blocking of collagen interactions.

### Inhibiting collagen synthesis

Collagen can be found in either its triple helical intact state or its unfolded denatured state. In fibrotic conditions such as cancer, there is an overproduction of intact collagens 59, 106. The most common approach to reduce collagen synthesis has been to inhibit TGF-β signaling and thus alter its regulatory role in collagen synthesis. Halofuginone inhibits type I collagen synthesis and has been shown to be effective in reducing fibrosis in murine pancreatic and liver cancer models 109, 110. Similar promising results were obtained in murine melanoma models 111. In 2011, the common anti-hypertensive drug losartan was repurposed to improve the efficacy of nano-formulations of drugs and viruses by inhibiting collagen synthesis 112. In a series of pre-clinical studies, losartan was administered by intraperitoneal injection to mice bearing human sarcoma or human melanoma tumor xenografts. Two weeks after losartan administration, mice were treated with either intravenous injection of PEGylated liposomal doxorubicin (Doxil) or intratumoral injection of oncolytic herpes simplex viruses 112. Losartan treatment has since been shown to be effective in other cancer models 113. Later, the clinical benefits of the anti-fibrotic effect of losartan were shown in a Phase II clinical trial testing a combination of losartan with the FOLFIRINOX (leucovorin, 5-fluorouracil, irinotecan, and oxaliplatin) chemotherapy regimen in pancreatic cancer (clinicaltrials.gov identifier: NCT01821729). The R0 resection rate (i.e., rate of conversion of unresectable to resectable tumor, mainly due to tumor shrinkage) was 61% among eligible participants. Moreover, 52% of treated patients had no detectable cancer cells after tumor resection 114, 115. Overall, the inhibition of TGF-β signaling has been shown to be effective in enhancing the penetration of small-molecule drugs and nano-formulations into tumors 116, 117. However, as suggested by Lampi et al., strategies involving growth factors such as TGF-β are not without their caveats, since growth factors can have a multifaceted impact on cell behavior beyond ECM cross-linking 33. Moreover, TGF-β is important for inflammatory regulation and can have both pro- and anti-tumorigenic effects in cancer 33. These paradoxical roles underscore the complexity of modifying the ECM by this approach and have thus encouraged the development of different classes of therapeutics for targeting specific components of the TGF-β pathway 118, 119.

### Degradation of stromal collagen

In the 1980s, patients suffering from severe back pain were treated using collagenase injections into their spinal discs (to dissolve excess collagen) 120. Xiaflex^®^, an injectable form of bacterial clostridium histolyticum collagenase, was approved for the treatment of Dupuytren's contracture, a condition that results in hand deformity 121. Additionally, collagenases are used clinically to improve the tissue healing process in burn injuries 122. Since collagen is the most prevalent component of tumor ECM, it is also an attractive therapeutic target in cancer therapy 123 and thus, collagenases are used to improve drug and nano-formulation penetration into tumors. In this context, several studies have investigated the concurrent and subsequent administration of nano-formulations with collagenase. In one study, the co-injection of collagenase along with oncolytic herpes simplex virus vector MGH2, directly into the tumor, resulted in enhanced and more homogenous distribution of the viral vector within tumors in a human xenograft model of melanoma 124. In another study, intravenous injection of type I collagenase improved gene expression of a cationic liposome/plasmid DNA complex (lipoplex) in a lung tumor xenograft model. It was shown, that the favorable accumulation of lipoplex was due to a decrease in the interstitial pressure within the tumor 125. In comparison to carboxylated 100 nm polystyrene nanoparticles, analogs decorated with collagenase penetrated four-fold more into the core of human cervical carcinoma spheroids *in vitro* 126. In a recent study, to enhance the extremely short half-life of collagenase in the circulatory system (i.e., minutes) 127 and increase its accumulation at the tumor site, a liposomal formulation of collagenase type-I (i.e., collagozome) was developed 128. Collagozome was shown to be effective in degrading collagen in mice bearing pancreatic tumors or with fibrotic livers. For instance, histological staining showed that collagen levels within the tumors were reduced 15% more with collagozome relative to treatment with free collagenase. Moreover, in a series of experiments, mice bearing pancreatic tumor xenografts were pretreated with intravenous injection of collagozome 24 hours in advance of treatment with a micelle formulation of paclitaxel. The combination therapy resulted in an 87% reduction in tumor size compared to mice pretreated with empty liposomes and paclitaxel micelles. Whereas only a 60% reduction in tumor size was achieved following administration of free collagenase and paclitaxel micelles in comparison to the empty liposome and paclitaxel micelle control 128. In addition to administering exogenous collagenase, an alternative approach is to administer relaxin, which stimulates the synthesis of collagenase and down-regulates collagen production 129, 130. Administration of relaxin promoted the enhanced penetration of fluorescent-labeled dextran into human osteosarcoma spheroids *in vitro* 131. Overall, multiple studies have shown that collagenase treatment leads to improved drug transport and penetration into tumors. For a detailed discussion on this topic, the reader is directed to a recent review by Dolor et al. 123.

The interaction of MMPs with collagen is another active area of research. Overall, 23 different MMPs that target different components of the ECM are known 132. MMPs are classified according to their proteolytic substrate. For instance, gelatinases (MMP-2 and -9) digest denatured collagen types IV, VII, and X and collagenases (MMP-1, -8, -13 and -18) cut intact triple-helical collagen I, II, and IV 86. Thus, there has been great interest in developing therapeutic strategies that influence MMP activity 133, 134.

Depletion of tumor collagen is not without its caveats. It can result in the release of bioactive molecules such as cytokines and growth factors embedded within the stromal ECM as well as the recruitment of inflammatory cells. This can lead to a cascade of immuno-inflammatory responses that can enhance tumorigenesis 135. Moreover, depletion of tumor collagen can enhance tumor invasion by facilitating the access of tumor cells to the blood stream 136, 137. Lastly, it may affect the efficacy of collagen-targeted nano-formulations, although the magnitude of the effect is unclear and warrants further investigation. Of note, degradation of collagen results in an abundance of denatured collagen that can be targeted by collagen mimetic peptides (CMP), which bind specifically to the latter 138. Thus, functionalization of the surface of nano-formulations with CMP may be a better approach when combining ECM normalizing therapies aimed at collagen removal with collagen targeted nano-formulations.

### Prevention of collagen cross-linking by inhibition of LOXs

Preventing collagen cross-linking is another therapeutic approach to reduce fibrosis. LOX activity is frequently elevated in tumors and results in stiffening of tissues 139. Reduction in LOX activity has been shown to reduce tissue stiffness, prevent fibrosis 140, and tumor progression in multiple tumor models 141, 142. For instance, LOX inhibition, using 1 mg/kg LOX-blocking antibody, in a mouse model of pancreatic cancer enhanced the efficacy of the anti-cancer drug gemcitabine 143. In another study, the functionalization of poly(lactide-*co*-glycolide) nanoparticles with a LOX inhibiting antibody to prevent breast tumor growth was investigated. The results revealed that LOX-targeted nanoparticles were more effective when compared to a soluble anti-LOX antibody (which was not accompanied by nanoparticles) *in vitro* in mouse mammary cancer cells and *in vivo* in a mouse breast cancer xenograft model 144. However, in spite of preclinical success, this approach may be of limited usefulness. As suggested by Dolor et al., this is because impeding matrix synthesis when a dense matrix is already formed is not beneficial. For instance, the combination of gemcitabine with simtuzumab (anti-LOXL2) in a Phase II trial in metastatic pancreatic cancer patients failed to show improvement in clinical outcomes (clinicaltrials.gov identifier: NCT01472198). This was attributed to the advanced stage of the cancers 123, 145. Of note, defects in either LOX activity 88, or absence of sites of LOX crosslinking (e.g., collagen telopeptides) have been shown to play a role in tumor invasion and metastasis 61.

### Blocking collagen and integrin signaling

Blocking collagen signaling is another anti-fibrotic therapeutic strategy, and many attempts have been made to disrupt the interactions between collagen and its partners. Integrins are the partner receptors of collagen, to which collagen binds and activates 59, 83. Integrins play an important role in fibrosis, and their inhibition has resulted in prevention of disease progression 78. Vedolizumab is an integrin inhibitor that is currently approved for Crohn's disease and ulcerative colitis. Vedolizumab binds exclusively to the α4β7 integrin of pathogenic gut-homing lymphocytes, and thus acts as a gut-selective anti-inflammatory biologic 146. Due to its clinical efficacy as an anti-inflammatory agent and the fact that persistent inflammation often leads to fibrosis, it can be construed that integrin-specific inhibitors may have the potential to be used therapeutically as anti-fibrotic agents. An overview of therapies based on integrins has recently been provided by Schnittert et al. 78.

## Hyaluronic acid as a therapeutic target to remodel ECM

As one of the major non-sulfated glycosaminoglycans, hyaluronic acid (also termed hyaluronate or hyaluronan) is another attractive component of the ECM to target in fibrotic stroma 100. Hyaluronic acid accumulation correlates with reduced elasticity and increased gelation pressure within tumor tissues 147. In fact, hyaluronic acid production has been shown to be increased in prostate cancer tumor spheroids exposed to high solid stress environments 148. High interstitial solid stress 31 can ultimately result in the collapse of blood vessels within the tumor tissue, leading to reduced accumulation of therapeutics 149. Thus, the degradation of hyaluronic acid in fibrotic stroma is expected to relieve solid stress. The therapeutic approaches aimed at influencing hyaluronic acid can be classified into three categories: (1) degradation of hyaluronic acid; (2) inhibition of hyaluronic acid synthesis; and (3) blocking hyaluronic acid signaling. The utility of blocking hyaluronic acid signaling has been reviewed elsewhere and will not be elaborated upon here 150. Of note, hyaluronic acid has been shown to act as a stromal tumor-suppressing factor. The reasons behind such a paradoxical effect remain to be explained. However, variability in hyaluronic acid metabolism and the regulation of its molecular weight are sugested as possible reasons 35.

### Degradation of hyaluronic acid

The delivery of hyaluronidase to degrade existing tumor hyaluronic acid has been explored in clinical trials in oncology since the 1980s 151, and improved outcomes in bladder, brain, gastrointestinal, and head and neck cancers, have been observed 123, 147. However, the bovine hyaluronidase administered in these studies caused immunogenic responses, which encouraged the development of a recombinant human hyaluronidase 152. For a detailed discussion of the utility of hyaluronidase for improving tumor penetration, readers are referred to comprehensive reviews on this topic 35, 147, 152-156. Currently, a PEGylated human hyaluronidase (PEGPH20) has entered late stage clinical trial evaluation. This polymer-modified formulation of recombinant hyaluronidase has reduced immunogenicity and prolonged circulation time as compared to unmodified native enzyme of non-human origin 156. A combination of PEGPH20 with gemcitabine in Phase I (clinicaltrials.gov identifier: NCT01453153) and gemcitabine/nab-paclitaxel in Phase II (clinicaltrials.gov identifier: NCT01839487) clinical trials have been successfully evaluated 157 and is now in Phase III clinical development (clinicaltrials.gov identifier: NCT02715804) (**Table 1**) 158. Surprisingly, in a parallel Phase II clinical trial where a combination of PEGPH20 with modified fluorouracil (FU), leucovorin, irinotecan, and oxaliplatin (mFOLFIRINOX) was evaluated (clinicaltrials.gov identifier: NCT01959139), the combination of PEGPH20 with mFOLFIRINOX worsened outcomes and reduced the overall survival by ~53%. Apparently, in this study the combination of PEGPH20 and mFOLFIRINOX resulted in increased toxicity that required reductions in dose and treatment duration of mFOLFIRINOX. Thus, the reduced overall exposure of patients to mFOLFIRINOX was likely a major contributor to the inferior outcomes in the PEGPH20/mFOLFIRINOX arm of the study 159, 160.

It is worth noting that hyaluronidase is overproduced in many types of cancer 100 and has been used to overcome issues faced by the delivery of polycationic agents (e.g., cationic cell penetrating peptides, chitosan, polyethyleneimine, and cationic lipids) to cancer cells, as discussed by Bernkop-Schnürch 161. In a fibrotic ECM with high expression of hyaluronic acid, the transport of drug delivery systems consisting of polycationic agents is impeded due to ionic interactions between the positively charged agents and negatively charged hyaluronic acid. To overcome this issue, many pre-clinical studies have shown improved transport of these systems by masking their positive charge through prior complexation with hyaluronic acid. In the presence of elevated levels of hyaluronidase at the tumor site, the degradation of the outer hyaluronic acid shell and subsequent release of the encapsulated polycationic cargo occurs 161.

### Inhibition of hyaluronic acid synthesis

Inhibitors of hyaluronic acid synthesis are used alone or in combination with hyaluronidase to enhance therapeutic efficacy 150. One of the inhibitors of hyaluronic acid is a compound known as 4-methylumbelliferone (4-MU). 4-MU is a coumarin derivative and was initially reported to suppress the synthesis of hyaluronic acid in cultured human skin 150, 162. 4-MU reduces hyaluronic acid synthesis by inhibiting hyaluronic acid synthases (HAS), and in mouse models 4-MU has been shown to reduce tumor progression 163, 164. Additionally, a combination therapy of liposomal doxorubicin with a liposome-encapsulated 4-MU prodrug improved overall survival in an orthotopic mouse model of breast cancer 165. This was attributed to the enhanced transport of the liposomal doxorubicin into the tumor tissue.

## Additional therapeutic strategies

Several other clinically-approved drugs have been investigated for their anti-fibrotic effects, including tranilast 166, pirfenidone 167, fasudil 168, metformin 169 and, dexamethasone 170. Clinical trials involving repurposed drugs such as hydroxychloroquine, defactinib, retinoic acid receptor agonists, macropinocytosis inhibitors, and focal adhesion kinase (FAK) inhibitors have recently been reviewed elsewhere 33, 171. As pointed out by Dolor et al., the results of the Phase III losartan trial (clinicaltrials.gov identifier: NCT01821729) will be a good indicator of whether or not modifications to tumor ECM using orally-bioavailable small molecules is feasible 114, 115, 123. Investigating the role of epigenetics in fibrosis is another active area of research 40. In this context, special attention has been paid to a subfamily of non-coding RNAs, named microRNAs, because of their important role in the wound healing response 172. Currently, several microRNA nano-formulations for targeted therapy of fibrotic diseases have shown potential for clinical development 173. However, there is still a pressing need to develop disease-specific and efficient microRNA carriers to improve diagnosis and treatment of fibrotic diseases.

**Figure 6:**
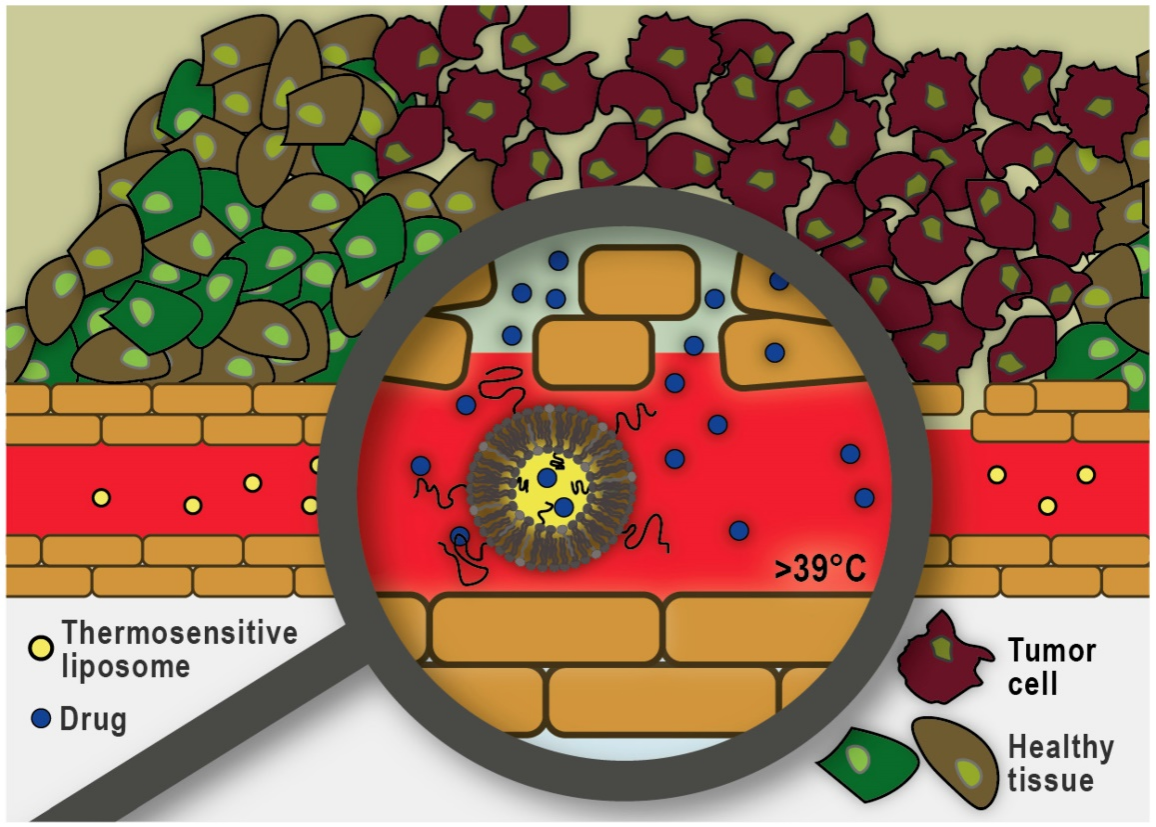
** Heat-triggered intravascular drug release from thermosensitive liposomes.** Localized mild hyperthermia is employed to heat the tumor area by a few degrees (39-43°C) which can trigger rapid drug release within the tumor microvasculature. The released drug enters the tumor interstitium via diffusion along the existing concentration gradient.

In addition to pharmacological modifications, the physical disruption of tumor microvasculature using focused ultrasound and microbubbles is also proving to be a promising strategy to improve nano-formulation transport into tumors. Ultrasound can cause the cavitation of circulating microbubbles resulting in localized shear stress on the surrounding vessels. This approach has been shown to result in increased accumulation of drugs and nano-formulations at the target site 174. Another exciting area of research focused on circumventing the dense ECM, as a significant barrier to nano-formulation transport, is the use of mild hyperthermia in combination with thermosensitive liposomes. As illustrated in **Figure 6**, this approach does not rely on the extravasation and distribution of the nano-formulation into the tumor interstitium. Instead, drug release is triggered within the tumor vasculature and subsequently the free drug molecules diffuse along the concentration gradient into the tumor interstitium. To trigger intravascular burst release, the target tissue is heated using focused, localized mild hyperthermia (39-43 °C) prior to administration of the liposomes. Heating is commonly continued for up to one hour to assure maximum drug release and accumulation at the target site 175. Thermosensitive liposomes loaded with doxorubicin (ThermoDox®) are currently being evaluated in a Phase III clinical trial in combination with radiofrequency ablation for the treatment of hepatocellular carcinoma (clinicaltrials.gov identifier: NCT02112656), as well as a Phase I clinical trial in combination with magnetic resonance guided high-intensity focused ultrasound for the treatment of pediatric refractory solid tumors (clinicaltrials.gov identifier: NCT02536183). An alternate strategy is to allow long-circulating thermosensitive liposomes to accumulate at the tumor site via the EPR effect and subsequently trigger extravascular release of the significantly smaller drug payload by applying localized mild hyperthermia 176. Overall, it has been shown that drug delivery using thermosensitive liposomes can result in increased drug accumulation and improved distribution throughout the tumor tissue and overcome many of the challenges previously reported with other nano-formulations that rely on passive targeting via the EPR effect.

## Imaging the ECM and monitoring ECM remodeling

Enhancing drug and nano-formulation transport into solid tumors by modifying their ECM has clinical potential. To this end, different combinations of nano-formulations and drugs with ECM remodeling effects have been investigated in cancers with fibrotic stroma such as pancreatic, ovarian, and lung cancers (**Table 1**). In addition to hyaluronidase and its PEGylated form (PEGPH20), for their use as ECM remodeling enzymes, other therapeutic agents such as paricalcitol, metformin, and nintedanib have been studied because of their potential anti-fibrotic effects. For instance, paricalcitol (a vitamin D analog) has been shown to be effective in reprogramming pancreatic stellate cells and restoring them to their non-activated phenotype. In normal pancreatic tissue, pancreatic stellate cells play an important role in ECM remodeling by producing ECM-degrading enzymes and ECM proteins 177. However, when they become activated, they acquire a myofibroblast-like phenotype and deposit abundant amounts of ECM 178. A reduction in ECM production by reprogramming the activated pancreatic stellate cells has also been documented for metformin (a glucose-lowering drug) 169. As for nintedanib, its anti-fibrotic and inhibitory effects on activated fibroblasts have been demonstrated in lung adenocarcinoma patients 179.

However, enhancing drug and nano-formulation transport into solid tumors by modifying their ECM is not without its caveats. For instance, this type of intervention may foster tumor cell migration and metastasis, compromising or even worsening outcomes. It is thus imperative to identify the appropriate pathological stage during which the implementation of such strategies is most appropriate. The duration and magnitude of the effects on the ECM are also important factors, due to the vastly different rates of turnover of ECM components. As such, time-dependent stromal changes should be monitored either by visualizing the ECM remodeling process with time, or by monitoring certain circulating biomarkers. For instance, several studies have compared injected hyaluronidases to collagenases for their ability to increase drug penetration into tumors. Overall, results showed that collagenases generally performed equal to- or better than hyaluronidases 123. However, differences in mechanism of action, safety, and the duration of the effect should be considered. For instance, as pointed out by Dolor et al., there are large differences between the degradation products produced by collagenase and hyaluronidase, as well as differences in rates of ECM turnover for collagen and hyaluronic acid. Hyaluronidase degrades linear hyaluronic acid into short oligosaccharides, while collagenase digests collagen into large fragments that may be difficult to isolate from collagen fibers (this only results in minimal changes of the collagen network structure on the macroscopic scale) 123. Hyaluronic acid has a rapid rate of turnover (days to weeks), while collagen's turnover is significantly slower (months to years) 123, 180. Thus, due to the slow recovery rate, potential changes within the collagen structure would have a profound effect on drug penetration. As such, it would be advantageous, if not necessary, to develop tools to image and monitor stromal ECM and ECM remodeling *in vivo*, as a means to optimize the interventions discussed in previous sections. Imaging of interactions between the tumor ECM and cancer-associated fibroblasts has been achieved with intravital imaging methods 181, but it would be beneficial to move towards non-invasive methods. A key barrier to the ability to measure efficacy of MMP inhibitors in clinical trials is the inability to monitor whether the inhibitors are reaching sufficiently high concentrations within the tumor tissue to perform their function. Non-invasive (or minimally-invasive) methods to image ECM remodeling would have been beneficial in a number of clinical trials 133, 134.

Within tumors, an abundance of denatured collagen can be found due to degradation of collagen by MMPs, which can be exploited for the purposes of imaging/diagnosis. Denatured collagen can be visualized with collagen mimetic peptides (CMP) that can bind to the latter. To monitor ECM-remodeling, CMPs have been tagged with a near infrared fluorophore and were shown to accumulate at denatured collagen sites in the tumor tissue of a xenograft model of human prostate cancer in mice 138. In a similar context, the functionalization of the surface of nanoparticles with collagen-binding molecules was investigated for improved imaging of the collagen matrix. For instance, high-density lipoprotein nanoparticles were labeled with a magnetic resonance contrast agent and the collagen-binding peptide EP3533, to monitor compositional changes in atherosclerotic plaques in a murine model of atherosclerosis regression 182. Alternatively, MMP-2 activity has been considered pre-clinically for detection of cancer using an MMP-2-responsive nanoprobe system, due to the overexpression of this enzyme in tumors. Such a system is commonly comprised of a quenched fluorophore that recovers its fluorescence upon digestion by MMP-2. In the absence of MMP-2, the fluorescence of this system is quenched, while the presence of high levels of MMP-2 in the tumor restores fluorescence. The *in vivo* imaging application of this system was demonstrated in xenograft models of human fibrosarcoma and glioma 183. In another interesting study evaluating MMP activity at the tumor site, a nanosensor for protease-activity was developed. The system was composed of thermosensitive liposomes that were coated with heat sensitive magnetic nanoparticles and encapsulated protease substrates. The use of an external magnetic field resulted in a localized increase in temperature that triggered the release of the encapsulated protease substrates from the thermosensitive liposomes. MMP present within tumor tissues degraded the substrates and the products of this reaction were detected by enzyme-linked immunosorbent assay (ELISA) 184.

Similar to collagen, hyaluronidase is over-produced in many types of cancer 100 and has been used as a diagnostic cue for high-grade bladder cancer 185. While this may suggest an incompatibility regarding the use of hyaluronidase for some types of cancer 147, 185, the high metabolism of hyaluronic acid due to elevated expression of hyaluronidase provides an opportunity for tumor detection/visualization. For instance, hyaluronic acid-tagged fluorescent gold nanoparticles have been used pre-clinically as a detection tool in metastatic ovarian cancer. When the surface-immobilized hyaluronic acid was cleaved by hyaluronidase, an increase in fluorescence signal was used to detect the cancerous tissues in a xenograft model of ovarian cancer in mice 186.

## Outlook

Overall, considering the complexity of the ECM, its variability within different tissues, and the multiple pathways by which homeostasis is maintained (in both normal and malignant tissues), the interventions discussed in this contribution produce inevitably complicated results. Although existing literature supports targeting ECM components as a promising therapeutic strategy, near depletion of stroma may compromise or even worsen the outcomes. This was shown in genetically- modified mouse models exhibiting a reduced stromal content. Both fibroblast-depleted mouse tumors 187 and sonic hedgehog-deficient tumors showed more aggressive pancreatic cancer behavior 188. Moreover, when a combination of a hedgehog inhibitor (IPI-926) with gemcitabine was evaluated in a Phase II clinical trial (ClinicalTrials.gov Identifier: NCT01130142), this combination resulted in a lower overall survival than what was historically achieved with gemcitabine alone 160. Excessive removal of ECM components may also result in tumor collapse and decreased drug penetration 18, 19, 72, 147, 155. Thus, regardless of the approach employed, normalization of the ECM rather than its depletion should be the primary goal. Of course, a substantial complementary effort must be made to develop formulations to transport the therapeutic agent(s) to the tumor, which may be anywhere in the body. This holds especially true as the interventions above need to be coordinated with additional drugs destined to act on either cancer cells and/or cancer-supportive cells (e.g., activated fibroblasts) as elegantly proposed by Daamen et al. 189. Notably, efforts to modify the ECM will be most beneficial to macromolecular therapeutics, since their transport is more sensitive to the ECM density than low molecular weight drugs 131. Similar beneficial effects can be obtained for immunotherapy where a dense desmoplastic stroma can act as a physical barrier to T-cell infiltration 190, 191. T-cells that infiltrate pancreatic cancers frequently become trapped in the dense stroma and do not contact tumor cells 192 and thus show lower sensitivity to immunotherapeutic agents such as immune checkpoint inhibitors 190.

**Figure 7:**
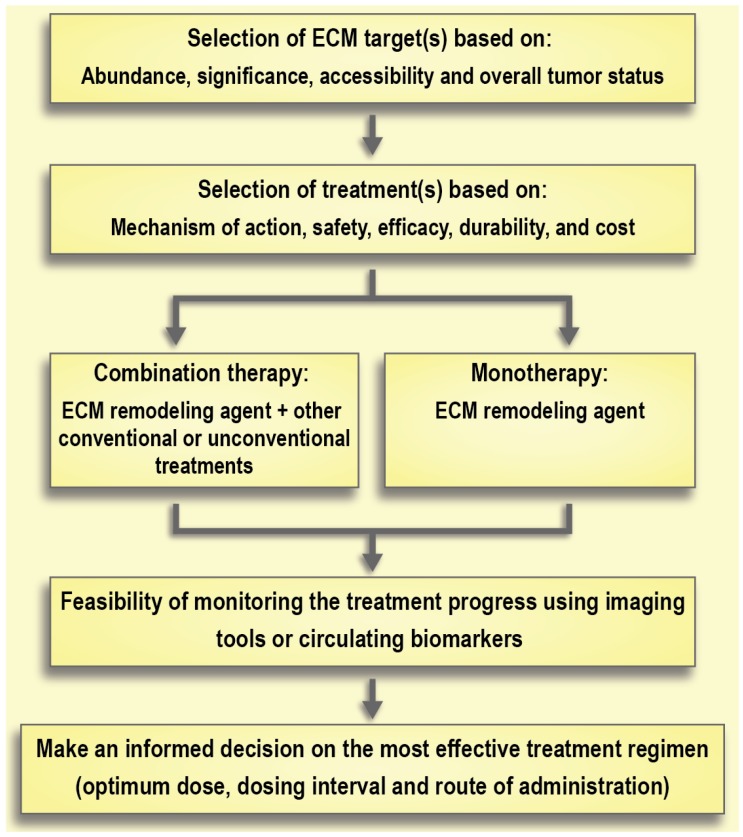
Schematic model of therapeutic interventions for normalization of the extracellular matrix (ECM) in solid tumors.

Selection of the treatment of choice based on its mechanism of action, safety, and durability of effects is of the utmost importance. For instance, there are large differences between the degradation products resulting from collagenase and hyaluronidase treatment (i.e., collagen fragments and hyaluronic acid, respectively). Hyaluronic acid has a rapid turnover (days to weeks), while collagen's turnover is significantly slower (months to years) 123, 180. This slow turnover of collagen generates safety concerns around the effect of removing collagen in healthy tissues. Moreover, bacterial collagenases can cause immune reactions because of their non-human origin, whereas PEGylated human hyaluronidases are already under investigation in clinical trials. Of note, long-term use of hyaluronidase is associated with some side effects. For instance, it can interfere with the process of wound healing or can cause thromboembolic and musculoskeletal events as observed in clinical trials 160. Aspects of timing or co-delivery will be a major technological challenge that should be addressed in parallel to research on ECM normalization (**Figure 7**). Indeed, the development of better imaging modalities to monitor the effects of therapeutic interventions on the ECM and ECM remodeling will contribute greatly to advancements in this field. Moreover, better preclinical models are needed to help close the gap between experimental results and clinical outcomes 193. Traditional 2-D cell cultures are not the preferred model for studying the effect of ECM modifications due to an absence of proper ECM structure 194. On the other hand, 3-D tumor spheroids may provide more reliable results since they recapitulate some features of the non-vascularized tumor such as an inhibitory ECM, epithelial tight junctions, and an outer proliferating region that surrounds intermediate layers of quiescent cells along with a necrotic core 195. Recent advances in the development of cancer organoids may also enable higher throughput *in vitro* assessment of therapeutic strategies to address the tumor ECM 196.

## Figures and Tables

**Table 1:** Overview of clinical trials investigating drugs with ECM remodeling properties in combination with nano-formulations for cancer therapy, as of July 17 2019

ECM remodeling drug	Design	Cancer type		Clinical Phase	Clinicaltrials.gov identifier
**Liposomal Irinotecan, Onivyde^ ®^**					
Paricalcitol	Liposomal Irinotecan + 5-FU + Leucovorin + Paricalcitol	Pancreatic cancer		1	NCT03883919
**Protein-bound paclitaxel, Nab-Paclitaxel (nanoparticle albumin-bound paclitaxel), Abraxane^®^**					
PEGPH20 (PEGylated hyaluronidase)	PEGPH20 + Nab-paclitaxel + Gemcitabine VS Nab-paclitaxel + Gemcitabine	Pancreatic cancer		2	NCT01839487
	PEGPH20 + Nab-paclitaxel + Gemcitabine + Rivaroxaban	Pancreatic cancer		N/A	NCT02921022
	PEGPH20 + Nab-Paclitaxel + Gemcitabine VS Placebo + Nab-Paclitaxel + Gemcitabine	Pancreatic cancer		3	NCT02715804
	PEGPH20 monotherapy followed by combination therapy of PEGPH20 + Nab-Paclitaxel + Gemcitabine	Pancreatic cancer		2	NCT02487277
					
Paricalcitol	Paricalcitol IV + Nab-paclitaxel + Gemcitabine VS Paricalcitol oral + Nab-paclitaxel + Gemcitabine VS Placebo + Nab-paclitaxel + Gemcitabine	Pancreatic cancer		1/2	NCT03520790
	Paricalcitol + Nab-paclitaxel + Gemcitabine + Nivolumab VS Nab-paclitaxel + Gemcitabine + Nivolumab	Pancreatic cancer		Early 1	NCT03519308
	Paricalcitol + Nab-paclitaxel + Gemcitabine + Cisplatin	Pancreatic cancer		2	NCT03138720
	Paricalcitol IV + Nab-paclitaxel + Gemcitabine	Pancreatic cancer		N/A	NCT02030860
	Paricalcitol + Nab-paclitaxel + Cisplatin + Gemcitabine	Pancreatic cancer		2	NCT03415854
	Paricalcitol IV + Nab-paclitaxel + Cisplatin + Gemcitabine + Nivolumab	Pancreatic cancer		2	NCT02754726
Nintedanib	Nintedanib monotherapy followed by combination therapy of Nintedanib + Gemcitabine + Nab-Paclitaxel	Pancreatic cancer		1/2	NCT02902484
Nintedanib	Nintedanib + Nab-Paclitaxel VS Placebo + Nab-paclitaxel	Non-small cell lung cancer		1/2	NCT03361319
Metformin	Metformin + Nab-Paclitaxel + Gemcitabine + Dietary supplement	Pancreatic cancer		1	NCT02336087
Hyaluronidase	VCN-01 (genetically modified human adenovirus encoding human PH20 hyaluronidase) + Gemcitabine + Nab-Paclitaxel	Pancreatic cancer		1	NCT02045589
Hyaluronidase	VCN-01+ Nab-Paclitaxel + Gemcitabine VS VCN-01	Pancreatic cancer		1	NCT02045602
**PEGylated Liposomal Doxorubicin (PLD), Doxil^®^, Caelyx^®^**					
Nintedanib (BIBF 1120)	Nintedanib + PLD + Carboplatin	Ovarian cancer, or peritoneal cancer		1	NCT01314105
	Nintedanib + PLD	Ovarian cancer		Terminated (funding withdrawn due to drug unavailability)	NCT01485874
	Nintedanib + PLD + Carboplatin	Ovarian cancer		Terminated	NCT01329549

ECM, extracellular matrix; 5-FU, 5-fluorouracil.

**Table 2:** Summary of ECM targeting strategies

Mechanism	Agent	Treatment objective	Pathological conditions
**Collagen**			
Inhibition of collagen synthesis via TGF-β signaling	Intraperitoneal injection 109 or oral administration 110 of Halofuginone	Reduce fibrosis	Murine models of pancreas 109 and liver fibrosis 110
	Intraperitoneal injection of Halofuginone	Inhibit the establishment and progression of melanoma bone metastases	Murine melanoma 111
	Oral administration of Losartan	Enhance the efficacy of FOLFIRINOX chemotherapy	Human pancreatic cancer (NCT01821729) 114
Degradation of stromal collagen	Intratumoral injection of collagenase	Enhance the distribution of a herpes simplex virus vector	Human melanoma xenograft 124
	Intravenous injection of collagenase	Improve the accumulation of a liposome/plasmid DNA complex	Murine lung tumor model 125
	Collagenase-functionalized polystyrene nanoparticles	Enhance the penetration of the nanoparticles in multicellular spheroids	Human cervical carcinoma multicellular tumor spheroids 126
Stimulation of collagenase synthesis and downregulation of collagen production	Relaxin	Enhance the penetration of fluorescent-labeled dextran	Human osteosarcoma spheroids 131
Binding to denatured collagen	Collagen mimetic peptides	Monitor ECM-remodeling	Human prostate cancer xenograft 138
Binding to intact collagen	High density lipoprotein nanoparticles decorated with collagen binding molecules	Imaging of exposed collagen network	Murine model of atherosclerosis regression 182
Inhibition of collagen cross-linking	Simtuzumab (anti-LOXL2)	Enhance the efficacy of combination therapy with gemcitabine	Pancreatic cancer (NCT01472198) 145
Inhibition of collagen cross-linking	Poly(lactide-co-glycolide) nanoparticles decorated with LOX inhibitory antibody	Reduce tumor growth	Breast cancer xenograft mouse model 144
Imaging MMP-overexpressing cells	Nanoprobe system with a MMP-labile linker	Image MMP-2-overexpressing tumors	Human fibrosarcoma and glioma xenografts 183
Binding to integrins	Nanoparticles decorated with integrin binding molecules	Enhance tumor treatment and imaging	Multiple models 78
**Hyaluronic acid**			
Degradation of hyaluronic acid	Intravenous infusion of PEGylated human hyaluronidase (PEGPH20)	Enhance the efficacy of combination therapy with gemcitabine and nab-paclitaxel	Human pancreatic cancer (NCT02715804) 158
Hyaluronidase substrate	Hyaluronic acid tagged-gold nanoparticles	Detect hyaluronidase-overexpressing tumors	Ovarian tumor xenograft 186
Hyaluronidase substrate	Complexation of hyaluronic acid and cationic agent	Enhanced tumor penetration of polycationic agents	Multiple models 161
Inhibition of hyaluronic acid synthesis	4-methylumbelliferone	Reduce tumor progression	Multiple cell lines (*in vitro*) 163, 164
Inhibition of hyaluronic acid synthesis	Liposome-encapsulated 4-methylumbelliferone	Enhance the efficacy of combination therapy with liposomal doxorubicin	4T1 murine breast tumor model 165

ECM, extracellular matrix; FOLFIRINOX, the FOLFIRINOX chemotherapy regimen is a combination of the drugs 5-fluorouracil, leucovorin and oxaliplatin; LOXL2, Lysyl oxidase‐like 2; MMPs, matrix metalloproteinases; TGF-β, transforming growth factor beta.

## Abbreviations

4-MU: 4-methylumbelliferone
ADK: adenosine kinase
ADAM: a disintegrin and metalloproteinase
ADAMTs: ADAMs with thrombospondin motifs
BM: basement membrane
CAF: cancer-associated fibroblast
CD44: cluster of differentiation 44
CMP: collagen mimetic peptides
ECM: extracellular matrix
ELISA: enzyme-linked immunosorbent assay
EMT: epithelial to mesenchymal transition
EndMT: endothelial to mesenchymal transition
EPR: enhanced permeability an retention
FAF: fibrosis-associated fibroblasts
FGF: fibroblast growth factor
FOLFIRINOX: chemotherapy regimen is a combination of the drugs 5-fluorouracil, leucovorin and oxaliplatin
GAG: glycosaminoglycan
HAS: hyaluronic acid synthases
HSC: hepatic stellate cell
IFP: interstitial fluid pressure
Lipoplex: liposome/plasmid DNA complex
LOX: lysyl oxidase
MMP: matrix metalloproteinase
MMPI: MMP inhibitor
MSC: mesenchymal stem cell
PDGF: platelet-derived growth factor
RHAMM: hyaluronic acid-mediated motility
RNA: ribonucleic acid
siRNA: small Interfering RNA
TAF: tumor-associated fibroblasts
TGF-β: transforming growth factor beta
TME: tumor microenvironment
VEGF: vascular endothelial growth factor
αSMA: α‑smooth muscle actin
